# Network Inference of Transcriptional Regulation in Germinating Low Phytic Acid Soybean Seeds

**DOI:** 10.3389/fpls.2021.708286

**Published:** 2021-08-31

**Authors:** Lindsay C. DeMers, Victor Raboy, Song Li, M. A. Saghai Maroof

**Affiliations:** ^1^School of Plant and Environmental Sciences, Virginia Tech, Blacksburg, VA, United States; ^2^National Small Grains Germplasm Research Center, Agricultural Research Service (USDA), Aberdeen, ID, United States

**Keywords:** phytic acid, *myo*-inositol phosphate synthase, multidrug-resistance protein ABC transporter, seed germination, gene regulatory network, unsupervised machine learning, abscisic acid signaling, phosphate homeostasis

## Abstract

The low phytic acid (*lpa*) trait in soybeans can be conferred by loss-of-function mutations in genes encoding *myo*-inositol phosphate synthase and two epistatically interacting genes encoding multidrug-resistance protein ATP-binding cassette (ABC) transporters. However, perturbations in phytic acid biosynthesis are associated with poor seed vigor. Since the benefits of the *lpa* trait, in terms of end-use quality and sustainability, far outweigh the negatives associated with poor seed performance, a fuller understanding of the molecular basis behind the negatives will assist crop breeders and engineers in producing variates with *lpa* and better germination rate. The gene regulatory network (GRN) for developing low and normal phytic acid soybean seeds was previously constructed, with genes modulating a variety of processes pertinent to phytic acid metabolism and seed viability being identified. In this study, a comparative time series analysis of low and normal phytic acid soybeans was carried out to investigate the transcriptional regulatory elements governing the transitional dynamics from dry seed to germinated seed. GRNs were reverse engineered from time series transcriptomic data of three distinct genotypic subsets composed of *lpa* soybean lines and their normal phytic acid sibling lines. Using a robust unsupervised network inference scheme, putative regulatory interactions were inferred for each subset of genotypes. These interactions were further validated by published regulatory interactions found in *Arabidopsis thaliana* and motif sequence analysis. Results indicate that *lpa* seeds have increased sensitivity to stress, which could be due to changes in phytic acid levels, disrupted inositol phosphate signaling, disrupted phosphate ion (Pi) homeostasis, and altered *myo*-inositol metabolism. Putative regulatory interactions were identified for the latter two processes. Changes in abscisic acid (ABA) signaling candidate transcription factors (TFs) putatively regulating genes in this process were identified as well. Analysis of the GRNs reveal altered regulation in processes that may be affecting the germination of *lpa* soybean seeds. Therefore, this work contributes to the ongoing effort to elucidate molecular mechanisms underlying altered seed viability, germination and field emergence of *lpa* crops, understanding of which is necessary in order to mitigate these problems.

## Introduction

The development and commercialization of low phytic acid (*lpa*) crops could represent one approach to enhanced management of phosphorus (P) in animal agriculture and to addressing mineral deficiency in humans. Seed phytic acid [*myo*-inositol-(1,2,3,4,5,6)-hexa*kis*phosphate] represents about 75% of seed total P. In the intestinal tract of non-ruminant animals, seed-derived dietary phytic acid chelates divalent cations, and the resulting salts are excreted. This can contribute to mineral deficiencies in monogastric animals and leads to high levels of excreted phosphorus, which pollute water systems (Brown and Solomons, 1991; Ravindran et al., 1995; Weaver and Kannan, 2001; Bohn et al., 2008; Raboy, 2009a,b). *Lpa* barley, maize, rice, soybean, and wheat lines have been developed, and their seeds are shown to increase phosphorus availability in poultry and swine and reduce phosphorus pollution from the subsequent waste (Larson et al., 1998, 2000; Raboy et al., 2000; Wilcox et al., 2000; Hitz et al., 2002; Guttieri et al., 2004; Shi et al., 2007). Despite these advantages, *lpa* crops have not been commercialized, as they often exhibit poor seed and seedling vigor, low stress tolerance, and reduced germination and emergence rates (Meis et al., 2003; Oltmans et al., 2005; Bregitzer and Raboy, 2006; Raboy, 2007; Colombo et al., 2020). These undesirable traits represent downstream effects of perturbation of phytic acid synthesis/accumulation and/or the inositol phosphate pathways that it is part of, since they have fundamental roles in various developmental, metabolic, and signaling pathways critical to plant function (Raboy, 2009a). Therefore, a fuller understanding of the molecular basis behind these negatives will assist crop breeders and engineers in successfully handling them.

Some *lpa* crops, such as barley *lpa1-1* and common bean *lpa*-280-10, exhibit good seed emergence and yield, demonstrating that development of *lpa* crops without adverse agronomic effects is possible (Bregitzer and Raboy, 2006; Campion et al., 2009). Furthermore, selection within an *lpa* line might yield progeny with improved germination and field emergence (Oltmans et al., 2005). For example, a soybean *lpa* mutation termed TW-1 had reduced field emergence and reduced viability following seed storage; however, these negative effects were reduced in a single-plant derived line isolated in TW-1 progeny termed TW-1-M (Yuan et al., 2017; Yu et al., 2019). Limiting the wide-scale development of high performing *lpa* crops is a poor understanding of the molecular basis of seed phytic acid content in relation to seed vigor, i.e., the properties defining a seed’s potential performance during germination and emergence (Hampton and TeKrony, 1995). Previous studies with barley and soybean have investigated the effect of the *lpa* trait on developing seeds and found differences in energy metabolism and phytohormone signaling, as well as regulatory components that may be responsible for these variations (Bowen et al., 2007; Redekar et al., 2015, 2017). Transcriptomic and proteomic analyses of germinating seeds were used to understand the molecular basis of the improvement of field emergence and seed viability observed in the soybean mutant TW-1-M as compared with its parental line TW-1 (Yuan et al., 2017; Yu et al., 2019). These studies revealed changes in gene transcripts and proteins involved in energy metabolism, phytohormone pathways, oxidation-reduction processes, and stress responses (Yuan et al., 2017; Yu et al., 2019).

Because seed germination is recognized as the most vulnerable period in a plant’s life cycle (Rajjou et al., 2012), it is important to understand how the process of germination is regulated. During germination, seeds undergo a massive metabolic transition in order to prepare for seedling growth; this is a highly coordinated and complex process, involving regulatory control over cellular and metabolic events (Bewley, 1997; Bove et al., 2002; Fait et al., 2006; Rajjou et al., 2012). Primary factors found to mediate germination include metabolism, phytohormone signaling, signal transduction components, and notably, transcription factors (TFs; Howell et al., 2009; Rajjou et al., 2012; Bellieny-Rabelo et al., 2016). Thus, it is important to consider the influence of regulatory interactions between genes, as a systematic understanding of the processes governing germination can offer insights into seed and seedling vigor. Advancements in high-throughput technology, such as RNA sequencing (RNA-seq), enable the collection and analysis of genome-wide expression data at a systems level (van Dijk et al., 2014). These data can then be used to construct a gene regulatory network (GRN), a graphical or mathematical representation of the causal relationships between genes regulating cellular functions in an organism (Hecker et al., 2009; Li et al., 2015; Banf and Rhee, 2017). The GRN’s connections, representing interactions between genes, are established by implementing inference methods on transcriptomic data. Hence, inferred GRNs consist of computationally predicted directed interactions between TFs and target genes, making GRNs an effective tool for identifying key regulatory and target genes involved in specific biological processes (Krouk et al., 2013; Haque et al., 2019).

Previously, a GRN analysis was performed to understand how the *lpa* trait may be affecting seed vigor by comparing the transcriptomes of developing seeds in low and normal phytic acid soybeans (Redekar et al., 2017). Differences were found in metabolism, defense responses, phytohormone signaling, and candidate TFs putatively regulating some of these processes. However, to construct a more complete profile of low and normal phytic acid seed transcriptomes and to identify differences between their regulatory networks, this study examines RNA-seq data from germinating seeds of low and normal phytic acid soybeans and infers multiple GRNs. The findings offer new information on the transcriptional regulation of germinating *lpa* soybean seeds and the perturbed biological processes in *lpa* seeds that may be important to successful germination.

## Materials and Methods

### Genetic Material

In this study, eight experimental lines were used – *1mlpa*, 1MWT, *2mlpa*, 2MWT, 2MWT-L, 2MWT-N, *3mlpa*, and 3MWT (Table 1). The three *lpa* lines (*1mlpa*, *2mlpa*, and *3mlpa*) contain one, two, or three mutations, respectively, in genes functioning in the phytic acid pathway. These genes are MIPS1 (Glyma.11G238800), encoding *myo*-inositol-3-monophosphate synthase (MIPS), which catalyzes the first step in the pathway to phytic acid, and MRP-L (Glyma.19G169000) and MRP–N (Glyma.03G167800), encoding multidrug resistance-associated protein ATP-binding cassette (ABC) transporters (henceforth called MRPs), which transport phytic acid for storage (Saghai Maroof et al., 2009). The eight experimental lines represent three distinct subsets of genotypes. The first genotypic subset, designated as the “Mips” subset, contains the *lpa* line “*1mlpa*” (*mips1* mutation) and the normal phytic acid line “1MWT” (no mutation). These lines are isogenic and were developed from a cross between the normal phytic acid line “Essex” (no MIPS1 mutation) and the *lpa* line “V99-5089” (*mips1* mutation; Saghai Maroof and Buss, 2011). This *mips1* mutation conferring the *lpa* trait is the result of a point mutation on chromosome 11 (Saghai Maroof and Buss, 2011). The second genotypic subset, designated as “MRP,” contains four near isogenic lines, the *lpa* line “2mlpa” (*mrp-l* and *mrp-n* mutations) and three normal phytic acid lines, “2MWT” (no mutation), “2MWT-L” (*mrp-n* mutation only), and “2MWT-N” (*mrp-l* mutation only). These lines were developed from a cross between the *lpa* lines “CX-1834” (*mrp-1* and *mrp-n* mutations) and V99-5089 (no MRP mutation). The mutations conferring the *lpa* trait in *2mlpa* and CX-1834 are the result of point mutations in the epistatically interacting loci, MRP-L, and MRP-N, on chromosomes 19 and 3, respectively (Wilcox et al., 2000; Walker et al., 2006; Saghai Maroof et al., 2009). Lastly, the final genotypic subset, designated as “Mips-MRP,” is composed of the *lpa* line “*3mlpa*” (*mips1*, *mrp-1*, and *mrp-n* mutations) and the normal phytic acid line “3MWT” (no mutation). These lines were developed from a cross between CX-1834 and V99-5089. Seeds from all eight lines were harvested in 2017 from a field in Blacksburg, VA and stored at 4°C until experimentation.

**Table 1:** Characteristics and classification of parental and experimental soybean lines.

Soybean lines	Genotypic class subset	Genotype	Phytic acid	Emergence	Stachyose	Sucrose	Cross
V99-5089	-	*mips1*/MRP-L/MRP-N	Low	Low	Low	High	Parent
CX-1834	-	MIPS1/*mrp-l*/*mrp-n*	Low	Low	Normal	Normal	Parent
Essex	-	MIPS1/MRP-L/MRP-N	Normal	Normal	Normal	Normal	Parent
*1mlpa*	Mips	*mips1*/MRP-L/MRP-N	Low	Low	Low	High	Essex x V99-5089
1MWT	Mips	MIPS1/MRP-L/MRP-N	Normal	Normal	Normal	Normal	Essex x V99-5089
*2mlpa*	MRP	MIPS1/*mrp-l*/*mrp-n*	Low	Low	Normal	Normal	CX-1834 x V99-5089
2MWT	MRP	MIPS1/MRP-L/MRP-N	Normal	Normal	Normal	Normal	CX-1834 x V99-5089
2MWT-L	MRP	MIPS1/MRP-L/*mrp-n*	Normal	Normal	Normal	Normal	CX-1834 x V99-5089
2MWT-N	MRP	MIPS1/*mrp-l*/MRP-N	Normal	Normal	Normal	Normal	CX-1834 x V99-5089
*3mlpa*	Mips-MRP	*mips1*/*mrp-l*/*mrp-n*	Low	Low	Normal	Normal	V99-5089 X CX-1834
3MWT	Mips-MRP	MIPS1/MRP-L/MRP-N	Normal	Normal	Normal	Normal	V99-5089 X CX-1834

### Seed Germination and Sampling

Tissue from each line was sampled at three stages of seed germination in biological triplicate with 10 seeds per sample. The germination stages used were mature dry seeds (stage 1), 8 h imbibed seeds (stage 2), and germinated seeds (defined as radicle emergence; stage 3). For stage 1, seeds were ground to a fine powder using a P14 mill (Pulverisette 14, Fritsch) and stored at −80°C until use. Seeds for stages 2 and 3 were sterilized for 2 min with a 10% hypochlorite + Tris solution, washed in DI water three times for 5 min, and dried overnight. The seeds were then germinated on germination plates with filter paper and DI water in the dark at 29°C. Once the appropriate stage was reached, seed coats and radicles were removed, and the tissue was flash frozen with liquid nitrogen and stored at −80°C until use. The tissue from these stages was ground to powder with mortar, pestle, and liquid nitrogen. Total RNA from all stages was extracted using the RNeasy Plant Kit with on-column DNase digestion and RLC buffer (QIAGEN, Hilden, Germany). RNA quality was determined by UV spectrophotometry (260 nm, NanoDrop1000, Thermo Fischer Scientific, Waltham, MA, United States) and RNA integrity numbers (RIN; BioAnalyzer, Agilent Technologies, Santa Clara, CA, United States). Samples with a RIN value > 8.0 and 260/280 ratios > 2.0 were submitted to Novogene (Sacramento, CA, United States) for mRNA sequencing. A total of 72 samples (eight lines × three germination stages × three biological replicates) were sequenced with HiSeq4000 (Illumina, San Diego, CA, United States) to acquire 30 million, 150 PE reads per sample.

### Transcriptomics Data Processing and Differential Gene Expression

Raw reads were trimmed and filtered using Skewer (version 0.2.2) to remove adapter sequences and low-quality reads and bases (<Q30; Jiang et al., 2014). Using STAR (version 2.5.2b), the cleaned reads were aligned to “Williams82,” the well-annotated soybean reference genome (Wm82.a2.v1, downloaded from Phytozome; Schmutz et al., 2010; Dobin et al., 2013). Transcript abundances were calculated from the mapping results using featureCounts (version 1.5.1; Liao et al., 2014). These results were subsequently used for differential expression analysis with DESeq2 (version 1.22.2) in R (version 3.5.1; Love et al., 2014). Comparisons were made to identify differentially expressed genes (DEGs) between *lpa* and normal phytic acid lines at each stage within each subset of genotypes. DEGs were defined as those with false discovery rate (FDR)-adjusted value of *p* < 0.01, log_2_ fold change > |1.0|, and base mean > 10. The DEGs between *lpa* and normal phytic acid lines at each stage within each subset of genotypes can be found in Supplementary Table S1. The RNA-seq data from this study is available at the NCBI Gene Expression Omnibus (GEO) repository as GSE172018.

### Transcriptional Network Construction and Inference

For each subset of genotypes, gene expression levels were normalized for all genes using variance-stabilizing transformation in DESeq2 (Love et al., 2014). The normalized expression was averaged across the three replicates, and then the averaged expression of the DEGs was used for clustering. Clustering analysis for each genotypic subset was performed independently. DEGs from each subset were clustered using Gaussian-finite mixture modeling with the R package, mclust (version 5.4.2), and the best performing models were determined using Bayesian Information Criteria (BIC; Schwarz, 1978; Scrucca et al., 2016). For both the Mips and Mips-MRP genotypic subsets, nine clusters were found, and five clusters were found for the MRP genotypic subset. Gene ontology (GO) enrichment analysis was performed on each gene cluster using GO annotations obtained from Soybase (Grant et al., 2010). Significantly enriched GO categories were identified using Fisher’s exact test with FDR < 0.05 (Supplementary Table S2; Fisher, 1992). DEGs encoding TFs were annotated with the plant TFDB (Jin et al., 2017).

For this study, separate network inferences were performed on the three subsets of genotypes using a computational pipeline developed previously (Redekar et al., 2017; DeMers et al., 2020). The pipeline implements the module network approach (Segal et al., 2003), in which genes are clustered into co-expression modules (gene modules) and then gene regulation is inferred between TFs and gene modules. The pipeline also incorporates multiple inference methods for improved robustness. In the Mips subset, 489 differentially expressed TFs were identified. In the MRP subset, 24 differentially expressed TFs were identified, while 340 were identified in the Mips-MRP subset. These TFs were used as putative regulators of the gene co-expression modules. Gene expression was averaged for each module, and the values, along with the TF expression values, were used to build an expression matrix. This matrix was then used to infer putative regulatory interactions between TFs and modules by applying five distinct network inference algorithms: ARACNE, CLR, LARS, partial correlation, and Random Forest (Schafer and Strimmer, 2005; Margolin et al., 2006; Faith et al., 2007; Huynh-Thu et al., 2010; Haury et al., 2012). These algorithms represent the top-performing, unsupervised network inference methods, according to the DREAM5 challenge found in the benchmark paper by Marbach et al. (2012).

### Validation of Network Inferred Interactions

The interactions predicted by the five network inference methods were validated by comparison to published interactions observed in *Arabidopsis* using DAP-seq and motif sequence analysis. For validation against the published *Arabidopsis* interactions, each network inferred TF-module interaction was expanded to TF-gene interactions by matching the TF putatively regulating the module to all genes assigned to the module. The soybean TF-gene interactions were converted to homologous *Arabidopsis* interactions by identifying homologous *Arabidopsis* genes for soybean gene coding sequences. Using BLAST with an E-value threshold of 1e-5, the top *Arabidopsis* gene hit was selected. The resulting homologous *Arabidopsis* interactions were then compared to the published DAP-seq interactions to identify matches. For validation by motif sequence analysis, the motif discovery tool, MEME, from Meme Suite (version 5.0.4) was used to identify enriched motif sequences among the genes in each module using the 1,000 bps flanking those genes’ 5′ end. The enriched motif sequences were then compared to motif sequences found in *Arabidopsis* with DAP-seq by employing the TomTom tool from Meme Suite (version 5.0.4). This allowed for the identification of TFs that may recognize and bind the discovered motifs in each module. The identified TFs were then compared to the TFs predicted to be module regulators.

## Results

In this study, RNA-seq was carried out on germinating seeds from low and normal phytic acid soybeans, and GRNs were inferred to identify disparities in transcriptional regulation. A total of eight experimental soybean lines from three genotypic class subsets were used, with each subset containing at least one normal phytic acid line and one *lpa* line. The *lpa* trait was conferred through various combinations of mutant MIPS1, MRP-L, and MRP-N genes.

### Differential Expression Analysis

For each of the three subsets of genotypes (Table 1), the *lpa* and normal phytic acid lines were compared to identify genes that were differentially expressed at each stage. In the Mips subset, 5,841 DEGs were found between *1mlpa* (*lpa*) and 1MWT (normal phytic acid). Using a set of four near isogenic lines, the number of DEGs in the MRP subset was limited to just 430. This number was obtained by designating genes as DEGs only if they were differentially expressed in each comparison of *2mlpa* (*lpa*) to the three normal phytic acid lines (2MWT, 2MWT-L, and 2MWT-N). Finally, in the Mips-MRP subset, 4,512 DEGs were found between *3mlpa* (*lpa*) and 3MWT (normal phytic acid). The DEGs for each genotypic subset can be found in Supplementary Table S1.

**Figure 1:**
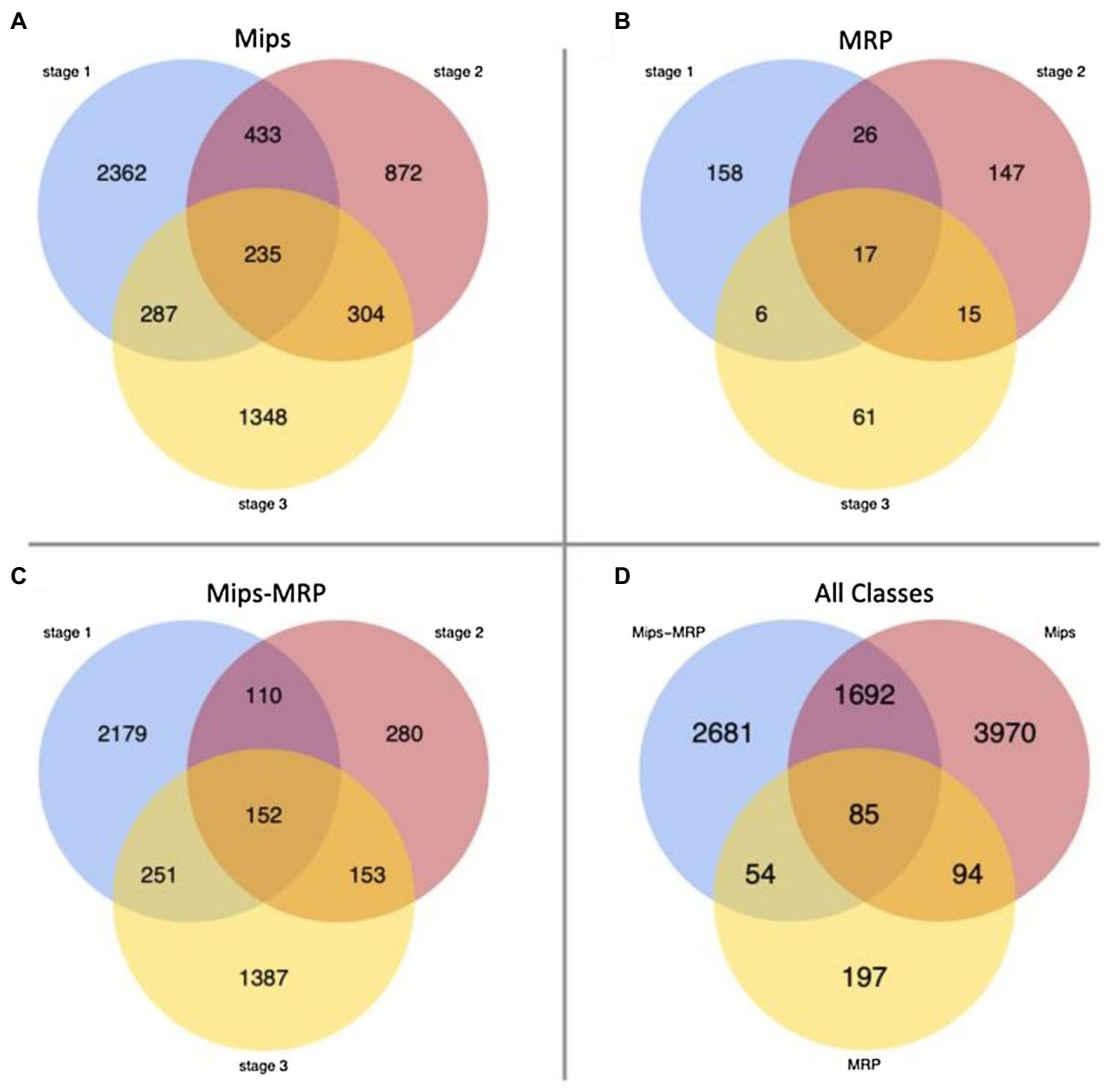
Venn diagrams of differentially expressed genes (DEGs). **(A)** Number of DEGs unique to and shared between each stage in the monophosphate synthase (Mips) subset. **(B)** Number of DEGs unique to and shared between each stage in the multidrug resistance-associated protein (MRP) subset. **(C)** DEGs in the Mips-MRP subset. **(D)** Number of DEGs unique to and shared between all three subsets of genotypes.

For each subset, few genes were differentially expressed in all three germination stages, indicating the mutations affect genes at specific stages (Figures 1A–C). In each subset, numerous genes were strictly differentially expressed at stage 1–40% in the Mips subset, 37% in MRP, and 48% in Mips-MRP (Figures 1A–C); this suggests both *mips1* and *mrp-1/mrp-n* mutations considerably affect genes at the dry seed stage. Not as many genes were differentially expressed in the Mips and Mip-MRP subsets at stage 2, indicating the *mips1* mutation may not impact genes as much in imbibed seeds. However, a substantial number were differentially expressed at stage 3 in germinated seeds (Figures 1A,C). Conversely, in the MRP subset, many genes were differentially expressed at stage 2, but few were differentially expressed at stage 3 (Figure 1B). This, along with the DEGs at germination stage 1, suggests the two *mrp* mutations have a greater effect on genes during the early stages of germination, at the dry and imbibed seed stages, when metabolism needs to be reinitiated.

When the DEGs from all three subsets were compared to one another, 85 genes were differentially expressed in all three genotypic subsets (Figure 1D). Roughly half of the DEGs in each subset remained unique to their particular subsets. However, there was a fair amount of overlap (1,692 DEGs) between the Mips and Mips-MRP subsets, which may be a result of shared perturbations in the *myo*-inositol synthesis pathway due to the *mips1* mutation. Less than 13% of the DEGs in the MRP subset were shared with those in the Mips-MRP subset, despite both of the subsets carrying the *mrp-l*/*mrp-n* mutations.

Both the Mips and Mips-MRP subsets had differential expression in genes functioning in the phytic acid biosynthesis pathway (Table 2). In each subset, Glyma.11G218500 and Glyma.18G038800, both encoding inositol 1,3,4-trisphosphate 5/6-kinase 4 (ITPK4), had increased expression in the *lpa* lines, *1mlpa* and *3mlpa*, predominantly in germination stage 1, the dry seed stage. In *1mlpa*, increased expression was also observed in two genes encoding inositol 1,3,4-trisphosphate 5/6-kinase 1 (ITPK1) and Glyma.11G238800, which had increased expression in all three germination stages and encodes *myo*-inositol-1-phosphate synthase 2 (MIPS2). These results suggest that mutantion in metabolic pathway gene (mips) has a strong effect on overall gene expression, whereas mutations in the transporter genes that are related to sub-cellular relocalization of nutrients have only mild effect on overall gene expression changes. This is signified by almost 20 times more genes that are differnetially expressed uniquely in mips mutant (3,970 genes, Figure 1D) than those are uniquely found in MRP mutants (197 genes, Figure 1D).

**Table 2:** DEGs in the phytic acid biosynthesis pathways.

Genotypic class subset	Gene ID	*Arabidopsis* homolog	Log2(FC) (*lpa*/Normal)			Gene symbol	Protein symbol
			Stage 1	Stage 2	Stage 3		
Mips	Glyma.11G238800	AT2G22240	2.5	1.6	1.2	MIPS2	*Myo*-inositol-1-phosphate synthase 2
Mips	Glyma.01G016700	AT5G16760	-	-	1.4	ITPK1	Inositol 1,3,4-trisphosphate 5/6-kinase 1
Mips	Glyma.09G206100	AT5G16760	1.0	-	-	ITPK1	Inositol 1,3,4-trisphosphate 5/6-kinase 1
Mips	Glyma.11G218500	AT2G43980	1.6	-	-	ITPK4	Inositol 1,3,4-trisphosphate 5/6-kinase 4
Mips	Glyma.18G038800	AT2G43980	1.3	-	-	ITPK4	Inositol 1,3,4-trisphosphate 5/6-kinase 4
Mips-MRP	Glyma.11G218500	AT2G43980	1.3	-	-	ITPK4	Inositol 1,3,4-trisphosphate 5/6-kinase 4
Mips-MRP	Glyma.18G038800	AT2G43980	2.3	1.1	1.1	ITPK4	Inositol 1,3,4-trisphosphate 5/6-kinase 4

### Co-expression Analyses Reveal Altered Phosphate Ion Homeostasis Activity and Stress Responses in *lpa* Lines

To compare the transcriptional regulation governing the dynamics of seed germination in *lpa* and normal phytic acid lines, gene co-expression modules were generated by individually clustering the set of DEGs found in each subset of genotypes. The co-expression modules, defined as sets of genes with similar temporal expression patterns, were created using a model-based clustering approach and BIC criterion. In the Mips subset, nine co-expression modules were found, five co-expression modules were found in the MRP subset, and nine were found in the Mips-MRP subset (Supplementary Table S1). GO analysis was carried out on each co-expression module for each genotypic subset. For the modules in the Mips subset, 372 instances of GO enrichment were found. In the MRP subset, 30 instances were found, and 162 were found in the Mips-MRP subset. These were narrowed down to focus on biological processes only. Enrichment for all categories (biological and molecular) can be found in Supplementary Table S2.

**Figure 2:**
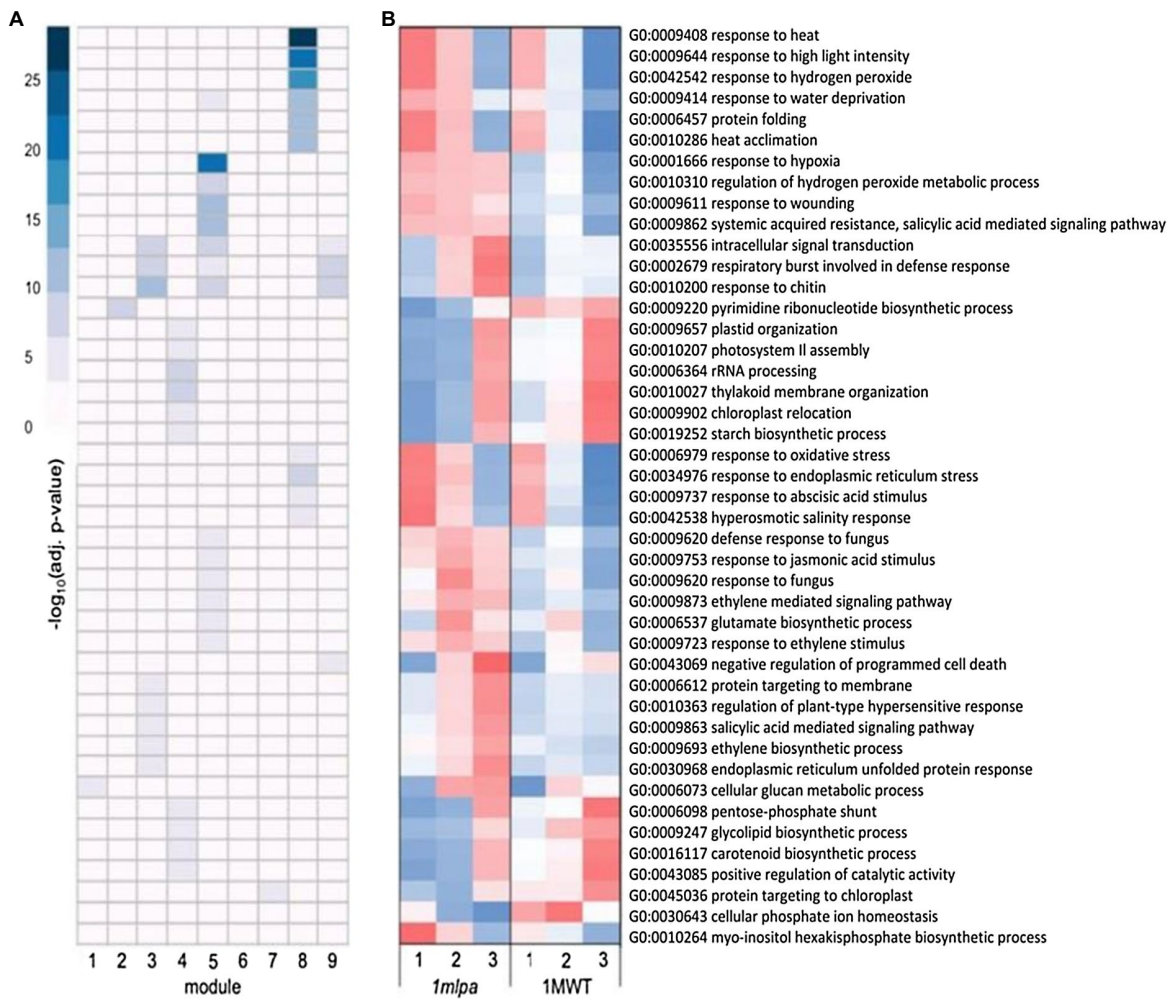
Monophosphate synthase subset gene co-expression modules and significantly enriched biological processes. A module is defined as a group of genes sharing similar expression profiles over time and likely involved in the same biological processes. Rows represent hierarchical clustering of significantly enriched gene ontology (GO) categories. Significantly enriched GO categories were defined as those with a false discovery rate (FDR) < 0.05. **(A)** Heatmap of significantly enriched GO biological processes in each gene co-expression module. Columns represent modules. Color represents −log_10_ adjusted value of *p*. **(B)** Average scaled expression of genes in significantly enriched biological processes. Columns represent germination stages of *1mlpa* and 1MWT. Red color represents increased expression, and blue color represents decreased expression. Enrichment for all GO categories can be found in Supplementary Table S2.

For the Mips subset co-expression modules, module 2 was enriched for nucleotide biosynthesis (Figure 2A), which had lower expression in *1mlpa* (Figure 2B). Modules 3, 5, and 9 were each enriched for signaling and stress-related processes, which were especially enriched in module 5 (Figure 2A). In all three modules, the genes functioning in the enriched processes had higher expression in *1mlpa* (Figure 2B). Modules 3 and 5 also showed enrichment for genes in the ethylene (ET) and salicylic acid (SA) pathways. For the enriched processes in module 4 (Figure 2A), many of which are related to photosynthesis, translation, and carbohydrate metabolism, gene expression was reduced in *1mlpa* in germination stages 1 and 2 (Figure 2B). In association with the phytic acid pathway, module 6 was enriched for *myo*-inositol hexa*kis*phosphate biosynthesis (GO:0010264), the genes of which had increased expression in stages 1 and 2 in *1mlpa* (Figure 2B). Conversely, in module 7, *1mlpa* had decreased expression in stages 1 and 2 in genes functioning in phosphate ion (Pi) homeostasis (GO:0030643; Figure 2B). Module 8 was enriched for genes in the abscisic acid (ABA) signaling pathway and was also strongly enriched for a number of stress-related processes, including response to heat, high light intensity, hydrogen peroxide, water deprivation, protein folding, heat acclimation, oxidative stress, and endoplasmic reticulum stress (Figure 2A). The genes in these processes exhibited increased expression in stages 1 and 2 in *1mlpa* (Figure 2B).

**Figure 3:**
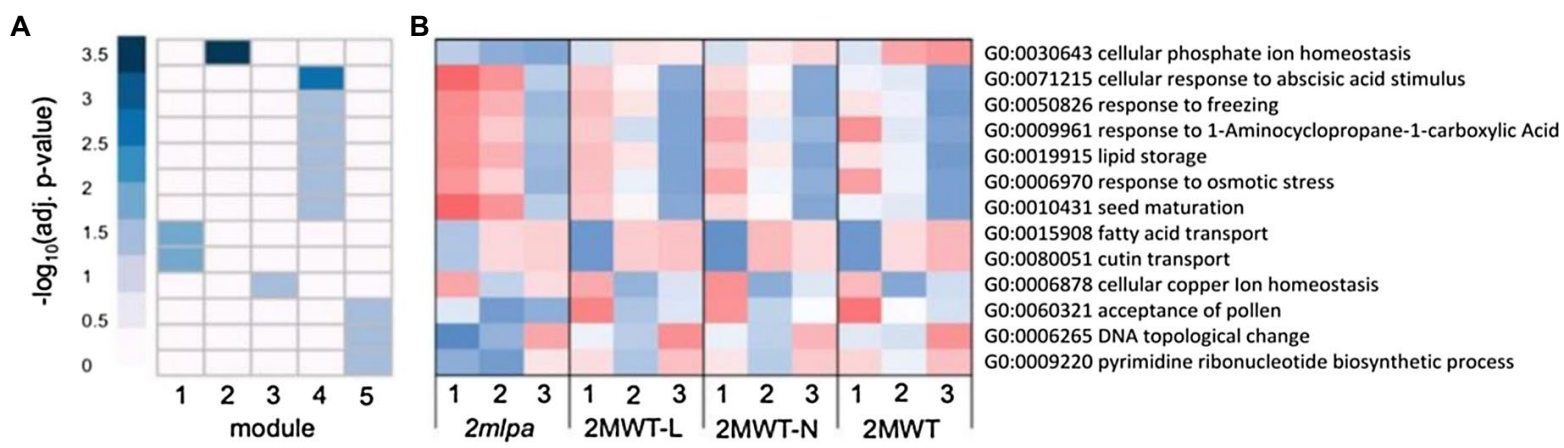
Multidrug resistance-associated protein subset gene co-expression modules and significantly enriched biological processes. A module is defined as a group of genes sharing similar expression profiles over time and likely involved in the same biological processes. Rows represent hierarchical clustering of significantly enriched GO categories. Significantly enriched GO categories were defined as those with an FDR < 0.05 **(A)** Heatmap of significantly enriched GO biological processes in each gene co-expression module. Columns represent modules. Color represents −log_10_ adjusted value of *p*. **(B)** Average scaled expression of genes in significantly enriched biological processes. Columns represent germination stages of *2mlpa*, 2MWT-L, 2MWT-N, and 2MWT. Red color represents increased expression, and blue color represents decreased expression. Enrichment for all GO categories can be found in Supplementary Table S2.

Each module in the MRP subset had strong gene enrichment in at least one biological process (Figure 3A). Figure 3B, an expression heatmap of the genes functioning in these processes, is especially interesting because it highlights the utility of the genetic material. That is, between the four isogenic lines, the three normal phytic acid lines (2MWT-L, 2MWT-N, and 2MWT) have nearly identical expression patterns, while expression in the single *lpa* line (*2mlpa*) is unique (Figure 3B). Module 1 was enriched for genes in fatty acid and cutin transport activities (Figure 3A) and at stage 1, had higher expression in *2mlpa* than the three normal phytic acid lines (Figure 3B). Module 2 was solely enriched for genes in cellular Pi homeostasis (GO:0030643), the genes of which had particularly reduced expression in germination stages 2 and 3 in *2mlpa*. In module 4, enrichment was found for genes functioning in ABA stimulus response, stress response, lipid storage, and seed maturation (Figure 3A). The genes in these processes had increased expression in *2mlpa* in germination stages 1 and 2 (Figure 3B). Lastly, module 5 had enrichment for nucleotide-related processes, the genes of which had decreased expression in stages 1 and 2 in *2mlpa* (Figures 3A,B).

**Figure 4:**
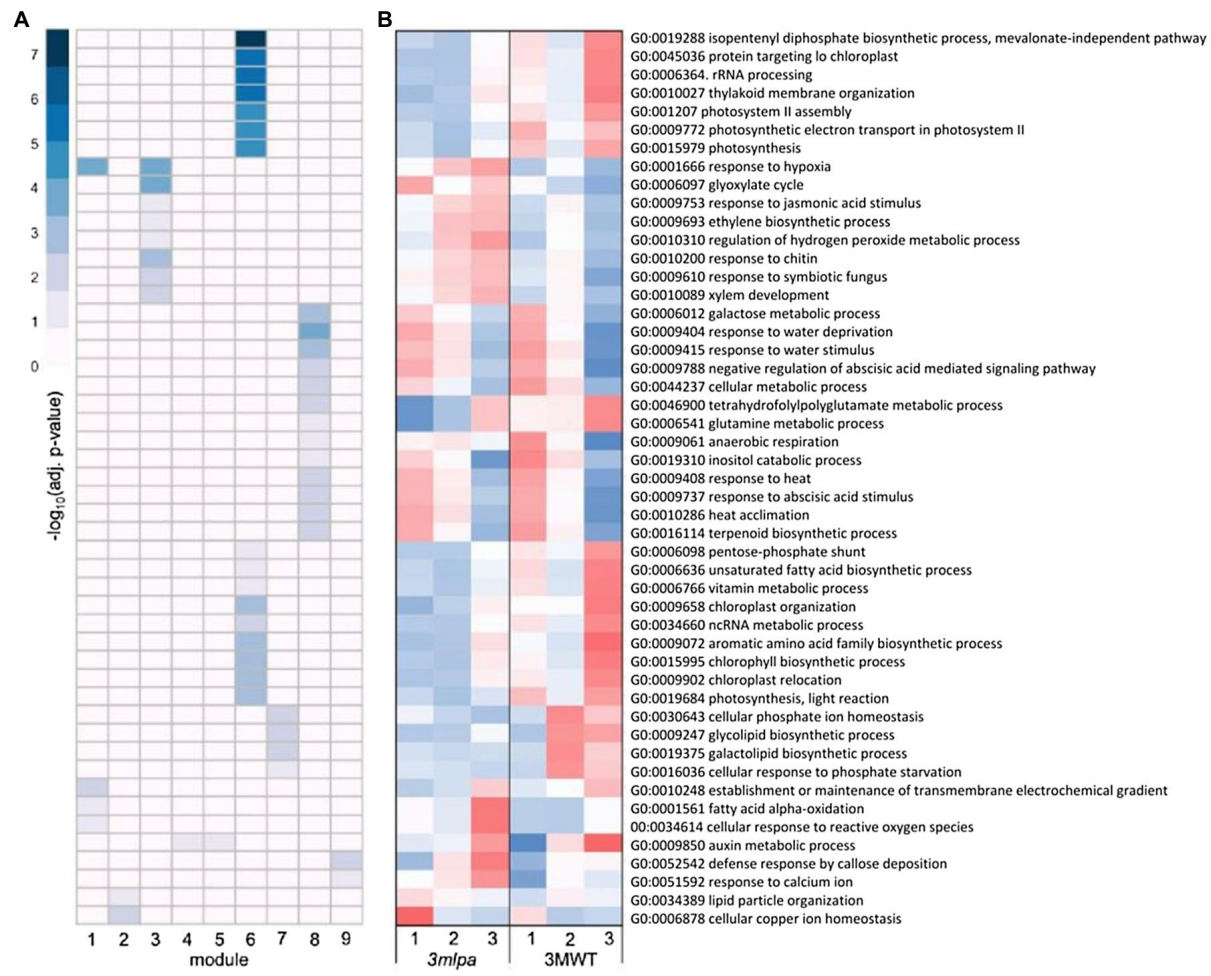
Mips-MRP subset gene co-expression modules and significantly enriched biological processes. A module is defined as a group of genes sharing similar expression profiles over time and likely involved in the same biological processes. Rows represent hierarchical clustering of significantly enriched GO categories. Significantly enriched GO categories were defined as those with an FDR < 0.05. **(A)** Heatmap of significantly enriched GO biological processes in each gene co-expression module. Columns represent modules. Color represents −log_10_ adjusted value of *p*. **(B)** Average scaled expression of genes in significantly enriched biological processes. Columns represent germination stages of *3mlpa* and 3MWT. Red color represents increased expression, and blue color represents decreased expression. Enrichment for all GO categories can be found in Supplementary Table S2.

For the Mips-MRP subset co-expression modules, module 1 was enriched for genes functioning in response to hypoxia and oxidative stress (Figure 4A), which had increased expression in *3mlpa* (*lpa*) in germination stages 2 and 3 (Figure 4B). Like the Mips and MRP genotypic subsets, the Mips-MRP subset had enrichment in module 3 for genes in stress responses as well as the glyoxylate cycle and phytohormone pathways involving JA and ET (Figure 4A). The genes in these processes had increased expression in *3mlpa* in stages 2 and 3 (Figure 4B). In the case of module 6 and similar to the Mips subset, strong enrichment was found for many genes in biological processes associated with photosynthesis, translation, and a number of metabolic pathways (Figure 4A). Like the Mips subset, gene expression for these processes was reduced in *3mlpa* in all three stages as compared to 3MWT (Figure 4B). Module 7 was enriched for genes functioning in glycolipid and galactolipid biosynthesis, Pi homeostasis, and response to Pi starvation (Figure 4A), which had decreased expression in *3mlpa* especially in stages 2 and 3 (Figure 4B). A number of biological processes were also enriched in module 8, such as several stress-related responses, ABA signaling, and other metabolic pathways (Figure 4A). Most of which had increased expression in *3mlpa* (Figure 4B).

When comparing GO enrichment between the three subsets of genotypes, the one biological process that was common between all of them was cellular Pi homeostasis (GO:0030643). Because *lpa* seeds have increased Pi levels, the significant expression changes observed in Pi homeostasis genes in germinating *lpa* seeds lends support to the putative role of phytic acid biosynthesis as a means of regulating cellular Pi concentration. Only seven genes in the soybean genome are annotated as functioning in cellular Pi homeostasis. The Mips subset had four DEGs from this GO category, the MRP subset had five DEGs, and the Mips-MRP subset had four DEGs. Between the three genotypic subsets, four DEGs were shared – Glyma.05G247900, Glyma.08G056400, Glyma.16G052000, and Glyma.19G098500. Both Glyma.05G247900 and Glyma.08G056400 encode purple acid phosphatase 17 (PAP17), and Glyma.16G052000 and Glyma.19G098500 encode glycerophosphodiester phosphodiesterase (GDPD1). The two PAP17 genes and the two GDPD1 genes were downregulated in all *lpa* lines at stage 2, and for the most part, they were also all downregulated at stage 3.

### Biological Processes Enriched in Both Developing and Germinating Seeds

In addition, the GO enrichment results for the Mips and Mips-MRP subsets were compared to the earlier enrichment findings in Redekar et al. (2017), where RNA-seq was performed on the same four experimental lines during seed development – *1mlpa*, 1MWT, *3mlpa*, and 3MWT. In the Mips subset, 88 GO categories overlapped with the developing seed expression data, and in the Mips-MRP subset, 41 categories overlapped. Both genotypic subsets had overlap in stress-, photosynthesis-, ion-, *myo*-inositol metabolism-, and hormone-related GO categories. Interestingly, overlap was also found in the pentose-phosphate shunt pathway (GO:0006098). In this study, the Mips subset had 64 genes in this pathway that were differentially expressed, and the Mips-MRP subset had 56. In both subsets, the genes were primarily differentially expressed at the dry seed stage (stage 1). This finding is notable because the pentose-phosphate shunt pathway parallels glycolysis, generating NADPH, pentoses (five-carbon sugars), and ribose 5-phosphate (precursors for nucleotide synthesis), but does so by oxidizing glucose-6-phosphate, the same substrate used by the MIPS enzyme in the first step of phytic acid biosynthesis (Kruger and von Schaewen, 2003).

### Gene Regulatory Networks

Inference of the constructed GRN detected TF-module interactions for each subset of genotypes. For each subset, the interactions were narrowed down to those detected by at least four out of the five inference methods (Supplementary Table S3). The TF-module interactions were then expanded into TF-gene interactions and computationally validated by comparison to the published *Arabidopsis* DAP-seq interactions and motif sequence analysis (O’Malley et al., 2016). For the Mips subset, this resulted in 4,572 TF-gene interactions, consisting of 31 differentially expressed TF regulators and 2,743 differentially expressed target genes (Supplementary Table S4). For the MRP subset, 154 TF-gene interactions were found, being regulated by five differentially expressed TF genes with 125 differentially expressed target genes (Supplementary Table S4). As for the Mips-MRP subset, 3,757 TF-gene interactions were found, which were regulated by 31 TFs and consisted of 1,998 target genes (Supplementary Table S4). Between the three subsets’ GRNs, the one putative TF regulator found in each was DREB1F encoded by Glyma.01G216000 (Table 3). This gene had increased expression in all three *lpa* lines, but no putative target genes of this TF were shared by all three subsets. Nonetheless, five other TF genes were identical in the Mips and Mips-MRP GRNs, with most of them sharing some of the same putative targets (Table 3). Interestingly, two of these genes (Glyma.04G249000, Glyma.06G114000) are homologous to the same TF, ATAF1, and both had increased expression in the *lpa* lines *1mlpa* and *3mlpa*. Though not shared in their networks, both *1mlpa* and *3mlpa* had an additional *ATAF1* gene with increased expression in their respective GRNs, Glyma.04G208300 in *1mpa* and Glyma.05G195000 in *3mlpa*. The changes in *ATAF1* expression in both *lpa* lines is notable as *ATAF1* is ABA-responsive and regulates ABA biosynthesis (Wu et al., 2009; Jensen et al., 2013).

**Table 3:** Putative candidate TFs shared between genotypic subsets’ GRNs.

TF gene	Arabidopsis homolog	TF family	Gene symbol	Subsets shared with	Log2(FC) (*lpa*/Normal)			Number of putative targets	Number of shared targets
					Stage 1	Stage 2	Stage 3		
Glyma.01G216000	AT1G12610	ERF	DREB1F, DDF2, ERF033	Mips	1.2	2.3	0	22	0
				MRP	0	2.6	2.1	26	
				Mips-MRP	2.5	2.6	1.9	32	
Glyma.02G080200	AT2G33710	ERF	-	Mips	0	0	1.4	103	5
				Mips-MRP	1.3	0	0	99	
Glyma.04G249000	AT1G01720	NAC	ATAF1, NAC2	Mips	0	0	1.8	365	12
				Mips-MRP	0	0	2.1	139	
Glyma.06G114000	AT1G01720	NAC	ATAF1, NAC2	Mips	0	1.2	1.9	158	31
				Mips-MRP	0	0	2.6	228	
Glyma.08G298200	AT2G02820	MYB	MYB88	Mips	0	0	1.1	117	0
				Mips-MRP	−1.1	0	1.3	149	
Glyma.16G167500	AT1G76890	Trihelix	GT-2	Mips	−2.0	0	0	114	11
				Mips-MRP	−1.2	−1.7	−1.3	115	

**Figure 5:**
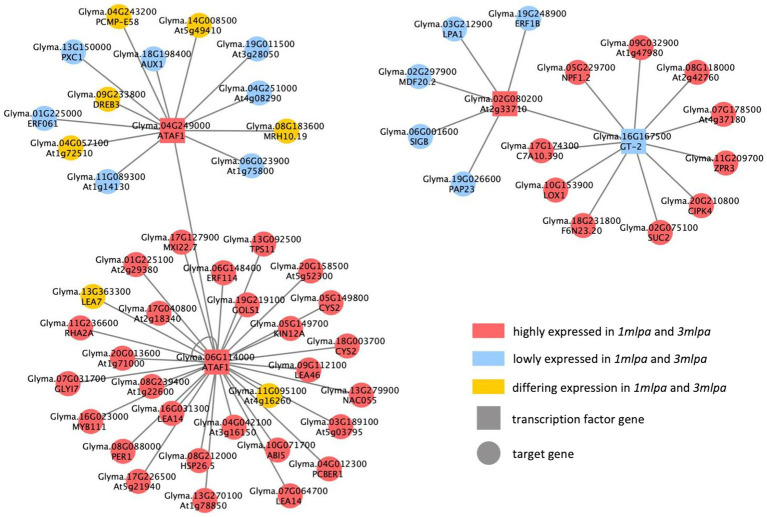
Consensus gene regulatory network (GRN) of Mips and Mips-MRP genotypic subsets. GRN of putative regulatory interactions between differentially expressed transcription factor (TF) genes and differentially expressed target genes found in both the Mips and Mips-MRP GRNs. TF genes (square nodes) directly regulate (gray edges) target genes (circular nodes). Nodes in red are genes with increased expression in *1mlpa* and *3mlpa*. Nodes in blue are genes with decreased expression in *1mlpa* and *3mlpa*. Nodes in yellow are genes with differing expression in *1mlpa* and *3mlpa*.

The Mips and Mips-MRP GRNs shared several of the same putative regulatory interactions (Figure 5). Some of the shared target genes that stand out include ABA-insensitive5 (*ABI5*; Glyma.10G071700) and multiple late embryogenesis abundant (*LEA*) genes (Glyma.07G064700, Glyma.08G239400, Glyma.09G112100, Glyma.13G363300, Glyma.16G031300, and Glyma.17G040800). *ABI5* is regulated by the ABA pathway and functions to retain embryos in a dormant state (Lopez-Molina et al., 2001). According to both networks, *ABI5* is putatively regulated by ATAF1 encoded by Glyma.06G114000, and in both *1mlpa* and *3mlpa*, the expression of *ABI5* is increased (Figure 5). All but one (Glyma.13G363300) of the six *LEA* genes had increased expression in *1mlpa* and *3mlpa*, and all are putatively regulated by *ATAF1* (Glyma.06G114000; Figure 5). This family of proteins is ABA-induced and reduces desiccation-induced cellular damage in seed tissue (Blackman et al., 1995).

As for the target genes in the MRP subset, seven were found in significant biological GO categories observed in the subset (Table 4). Most of these targets are seed storage proteins, which function in lipid storage, seed maturation, and responses to ABA stimulus. These seed storage proteins are putatively regulated by Glyma.05G032200 (MYB-related) and Glyma.07G060400 (bZIP), the latter of which encodes G-box binding factor 3 (GBF3).

**Table 4:** Putative target genes in the MRP subset with annotations for observed significant GO categories validated by *Arabidopsis* DAP-seq dataset and motif sequence analysis.

Gene name	*Arabidopsis* homolog	Gene symbol	Description	GO term	GO description(s)
Glyma.01G119600	AT2G40170	-	AT2G40170 protein	GO:0019915 GO:0050826	Lipid storage Response to freezing
Glyma.04G085000	AT4G32880	HB8	Homeobox-leucine zipper protein ATHB-8	GO:0010431	Seed maturation
Glyma.07G190100	AT1G61340	-	F-box protein	GO:0009961 GO:0006970	Response to 1-aminocyclopropane-1-carboxylic acid Response to osmotic stress
Glyma.10G037100	AT5G44120	CRA1, 12S STORAGE PROTEIN, CRU1	RmlC-like cupins superfamily protein	GO:0010431 GO:0071215	Seed maturation Cellular response to abscisic acid stimulus
Glyma.13G123500	AT1G03880	CRB, CRU2, CRU3	12S seed storage protein CRB (Cruciferin 2)	GO:0019915 GO:0071215 GO:0010431 GO:0050826	Lipid storage Cellular response to abscisic acid stimulus Seed maturation Response to freezing
Glyma.19G164900	AT5G44120	CRA1, 12S STORAGE PROTEIN, CRU1	RmlC-like cupins superfamily protein	GO:0010431 GO:0071215	Seed maturation Cellular response to abscisic acid stimulus
Glyma.20G148300	AT3G22640	PAP85	Vicilin-like seed storage protein (Glubulin)	GO:0019915 GO:0050826	Lipid storage Response to freezing

## Discussion

This study contributes to an elucidation of the key downstream effects on seed germination resulting from perturbation of seed phytic acid synthesis and storage.

### Regulation of Phosphate Ion Homeostasis in *lpa* Lines

Cellular phosphorus homeostasis is critical for normal seed development, maturation and subsequent germination (Theodorou and Plaxton, 1993). In all three *lpa* lines, enrichment was found for genes functioning in Pi homeostasis, indicating that the blocks in the phytic acid pathways do perturb cellular Pi homeostasis.

In the three *lpa* lines, Pi homeostasis enrichment was due to the same four genes – two genes encoding PAP17 and two genes encoding GDPD1. The two PAP17 and the two GDPD1 genes were downregulated in all three *lpa* lines at germination stage 2 and for the most part, were also downregulated at stage 3. Purple acid phosphatase (PAP) proteins are multifunctional proteins induced under Pi starvation and catalyze the hydrolysis of Pi from monoesters and anhydrides for the transport and recycling of Pi (Veljanovski et al., 2006). In particular, PAP17 also has peroxidation activity, functioning in the metabolism of reactive oxygen species (del Pozo et al., 1999). GDPD1 hydrolyzes glycerophosphodiesters and is also induced by Pi starvation, during which it likely releases Pi from phospholipids (Cheng et al., 2011). According to regulatory network inference and motif sequence analysis, all four Pi homeostasis genes are putatively regulated by Glyma.08G092300, which encodes a C2H2 TF. This TF may in part be responsible for downregulating Pi homeostasis genes in *lpa* lines. Downregulation of PAP17 and GDPD1 in *lpa* seeds suggests that sufficient, if not more than sufficient, cellular Pi levels are present. The increased Pi levels in *lpa* seeds may perturb Pi homeostasis in such a way that normal cell metabolism is disrupted, inducing cellular stress and ultimately reducing seed viability. Thus, reducing PAP17 and GDPD1 expression in *lpa* seeds may be an attempt to recover Pi homeostasis.

### Downstream Effects of Perturbed *Myo*-Inositol Metabolism in *mips1* Mutants

Phytic acid biosynthesis requires a substrate supply of *myo*-inositol and phosphate. The sole source of *myo*-inositol comes from the activity of the enzyme MIPS. Previous studies not just limited to soybean have also shown that loss-of-function mutations in MIPS1 are associated with impaired seed and plant performance (Meis et al., 2003; Pilu et al., 2003; Nunes et al., 2006; Obendorf et al., 2009; Donahue et al., 2010). Given that *myo*-inositol synthesis *via* MIPS is considered a part of general housekeeping (Chiera et al., 2004; Raboy, 2009a), it is not surprising that perturbing its expression can be detrimental or even lethal in some cases (Raboy, 2009a). In fact, MIPS2 (Glyma.11G238800) expression was increased in *1mlpa* in all three germination stages, perhaps in an attempt to restore the *myo*-inositol pool. In *lpa* lines *1mlpa* and *3mlpa*, increased expression was observed in ITPK1- and ITPK4-encoding genes as well, which interestingly are the ITPKs perturbation of which was demonstrated to reduce phytic acid in *Arabidopsis* mutants (Kim and Tai, 2011; Kuo et al., 2018). Also unique to the *mips1* mutation were significant expression changes in PIP5K encoding genes. These enzymes function in inositol pyrophosphate synthesis by phosphorylating InsP_7_ (derived from phytic acid) to InsP_8_. Changes in their gene expression is significant because of inositol pyrophosphates’ recognition as “energetic signaling” molecules, with roles in energetic metabolism, hormone signaling, and Pi sensing (Freed et al., 2020).

In the germination GRNs from this study and the developing seed GRN from the previous study (Redekar et al., 2017), the *lpa* lines carrying the *mips1* mutation (*1mlpa* and *3mlpa*) were significantly enriched for numerous stress responses. Genes encoding proteins functioning in these stress responses had increased expression in the *lpa* lines, indicating that *1mlpa* and *3mlpa* seeds have increased stress, thus impairing their viability and performance and ultimately reducing germination and emergence. Disruption of the *myo*-inositol metabolic pathway may have a negative effect on seed viability due to *myo*-inositol’s multifunctional nature in plant metabolism. In fact, several such effects that were found in this study are in accordance with the roles of *myo*-inositol (Loewus et al., 1973; Murthy, 1996; Obendorf, 1997; Peterbauer et al., 1998; Peterbauer and Richter, 1998; Slovin et al., 1999; Loewus and Murthy, 2000). For example, significant enrichment was found for genes functioning in the auxin pathway, cell death, cell wall metabolism, stress processes, and other carbohydrate metabolic pathways requiring *myo*-inositol as a precursor. In the case of stress, the increased expression of stress-related genes in *1mlpa* and *3mlpa* may in part be due to *myo*-inositol’s role as a substrate for the biosynthesis of raffinose, galactopinitol, and *O*-methyl inositols, which participate in stress-related responses and seed desiccation tolerance (Horbowicz and Obendorf, 1994; Obendorf, 1997; Peterbauer et al., 1998; Peterbauer and Richter, 1998). Therefore, changes in the contents of these compounds may alter *lpa* seeds’ ability to tolerate stress and desiccation. In additional support, MIPS1 is also required for cell death suppression (Donahue et al., 2010), and 145 and 80 genes involved in cell death were differentially expressed in *1mlpa* and *3mlpa*, respectively, suggesting cell death regulation is abnormal in these lines. The examples presented here demonstrate that depletion of the *myo*-inositol pool impact pathways that may affect seed viability in *1mlpa* and *3mlpa*. Thus, due to its many roles, perhaps MIPS1 is not the best target for conditioning the *lpa* phenotype in crop seeds.

### *Myo*-Inositol Metabolism and Seed Storage Proteins in *mrp-1*/*mrp-n* Mutant

Following synthesis, phytic acid and the mineral cations it chelates are transported into protein storage vacuoles (Lott et al., 1995). MRP proteins from the ABC transporter family are responsible for phytic acid transport and accumulation, as loss-of-function of these transporters can result in the *lpa* trait (Shi et al., 2007; Nagy et al., 2009; Saghai Maroof et al., 2009; Xu et al., 2009; Panzeri et al., 2011). Shi et al. (2007) hypothesize that phytic acid is not transported for storage but instead hydrolyzed in the cytoplasm by endogenous phytases in such *mrp* mutants, thereby preventing phytic acid accumulation. This is supported by concomitant increases in inositol intermediates and *myo*-inositol content (Shi et al., 2007; Israel et al., 2011). With elevated *myo*-inositol levels, it would be expected for other metabolic pathways utilizing *myo*-inositol to also be affected. Accordingly, *2mlpa* has increased expression in genes functioning in inositol trisphosphate metabolism, phosphatidylinositol transport, and *myo*-inositol transport. Alterations in inositol trisphosphate metabolism are noteworthy because it implies signal transduction is abnormal in *2mlpa*. One such gene that was upregulated was Glyma.17G219300 encoding a G-protein coupled receptor 1 (GCR1). G-protein coupled receptors function in the phosphatidylinositol signaling pathway, ultimately yielding two significant signaling molecules: inositol 1,4,5-trisphosphate (IP3) and diacylglycerol (DAG; Gilman, 1987). Consequently, the GCR1 encoding gene along with the other DEGs in inositol trisphosphate metabolism suggest irregular signaling may be a feature of *2mlpa*, all of which is a result of an elevated *myo*-inositol pool.

In *2mlpa*, increased expression was found in a number of genes encoding seed storage proteins. Many of these genes encode vicilin-like seed storage proteins and RmlC-like cupin 12S storage proteins. Seed storage proteins have particular importance because they provide an amino acid reserve for use during germination and seedling growth (Shewry et al., 1995). Whether an increase in protein storage content is a detriment to seed vigor is unclear. According to this network analysis, two genes were responsible for the upregulation of these genes’ expression – Glyma.05G032200 and Glyma.07G060400, encoding an MYB-related TF and GBF3, respectively. The induction of these TFs and the seed storage protein genes they putatively regulate is discussed further below.

### Altered Regulation in Auxin and ABA Signaling in *lpa* Seeds

Both phytic acid and *myo*-inositol are critical for normal auxin signaling. Phytic acid itself is in fact a cofactor of transport inhibitor response 1 (TIR1), an auxin receptor and primary mediator of auxin-regulated responses (Tan et al., 2007), and *myo*-inositol is essential for proper auxin transport and localization (Luo et al., 2011). Hence, it should not be surprising that auxin physiology was affected in the *lpa* mutants *1mlpa* and *3mlpa*, where 163 auxin-related genes were differentially expressed between *1mpa* and 1MWT and 155 differentially expressed between *3mlpa* and 3MWT. This finding is consistent with the previous seed development study using the same soybean lines (Redekar et al., 2017). Auxin signaling is a requirement for seed dormancy and germination inhibition but is so because it functions to enhance ABA action (Liu et al., 2013). In the current study, genes were identified encoding AUXIN RESPONSE FACTOR 10 (ARF10), an element that mediates crosstalk between auxin and the ABA signaling pathway during germination (Liu et al., 2013). These ARF10 genes, Glyma.13G325200 in *1mlpa* and Glyma.12G076200 and Glyma.13G325200 in *3mlpa*, had increased expression, which could affect the branch of the ABA pathway regulating germination in these lines. In fact, 345 and 209 ABA-related genes were differentially expressed in the *1mlpa* and *3mlpa* lines, respectively. Not only was the ABA pathway affected in this study, but it was also affected in transcriptome and proteome studies of germinating *lpa* soybeans also carrying *mips1* mutations (Yuan et al., 2017; Yu et al., 2019). This is significant as ABA is the sole hormone known to trigger and maintain seed dormancy and is a major inhibitor of seed germination (Cutler et al., 2010; Hubbard et al., 2010). Among the differentially expressed ABA genes identified in this study, Glyma.10G071700 and Glyma.13G153200 had increased expression in *1mlpa* and *3mlpa* and encode the bZIP TF ABI5. ABI5 reactivates late embryogenesis programs and arrests embryo growth during germination, causing the embryo to go into a state of dormancy. This is an adaptive response to environmental stress mediated by ABA with ABI5 functioning to maintain the quiescent state (Lopez-Molina et al., 2001). Thus, increased expression of ABI5 in *1mlpa* and *3mlpa* could promote seed dormancy and interfere with the embryos’ ability to resume growth.

The GRN from this study predicted two other TF genes, Glyma.04G24900 and Glyma.06G114000, as putative regulators of ABA-related genes in both the Mips and Mips-MRP genotypic subsets. Interestingly, these two TF genes are paralogs corresponding to the same *Arabidopsis* homolog, ATAF1, a TF whose transcript expression is induced in response to ABA and functions to positively regulate ABA biosynthesis (Jensen et al., 2013). In *Arabidopsis* ATAF1 overexpression studies, Wu et al. (2009) found that increased ATAF1 expression confers ABA hypersensitivity, oxidative stress hypersensitivity, and interferes with plant development. Both ATAF1-encoding genes were upregulated in *1mlpa* and *3mlpa*. Therefore, increased ATAF1 expression in *1mlpa* and *3mlpa* could contribute to the induction of ABA-responsive gene expression and thus irregularities in ABA signaling, such as upregulation of genes encoding ABI1, ABF2, AFP2, and PP2CA and downregulation of those encoding ABI4. Such expression disparities in prominent genes of the ABA signaling pathway could have serious effects on *1mlpa* and *3mlpa* seeds’ potential to complete germination and have normal seedling growth. Consistent with this study’s findings, Donahue et al. (2010) also observed impaired germination and increased ABA sensitivity during germination in *Arabidopsis mips1* mutants.

Despite carrying mutations affecting a different aspect of the phytic acid pathway, *2mlpa* from the MRP genotypic subset also had enrichment for stress responses and abnormalities in ABA signaling. Several of the ABA-related genes that were differentially expressed in *2mlpa* were seed storage proteins, the content of which is influenced by ABA, with high ABA levels stimulating their induction (Bray and Beachy, 1985; Finkelstein et al., 1985; Kagaya et al., 2005). According to their *Arabidopsis* homologs, ABA stimulus also induces expression of the two TF genes found to regulate the seed storage protein genes (Lu et al., 1996; Yanhui et al., 2006). These TF genes, the MYB-related TF encoded by Glyma.05G032200 and GBF3 encoded by Glyma.07G060400, had significantly increased expression in *2mlpa* in all three germination stages. Increased expression of the two TF genes and the storage proteins they putatively regulate suggests ABA levels are increased in the seeds of *lpa* line *2mlpa*. Correspondingly, GCR1 (Glyma.17G219300), which was upregulated in *2mlpa*, is a regulator of ABA signaling and has demonstrated involvement in seed dormancy according to its *Arabidopsis* homolog (AT1G48270; Chen et al., 2004; Pandey and Assmann, 2004). Thus, like *1mlpa* and *3mlpa*, the observed expression changes of ABA-related genes in *2mlpa* could promote seed dormancy and thereby inhibit germination.

Though different components of the ABA signaling pathway were affected, it is notable that the pathway was disrupted in all three *lpa* lines and that it was also disrupted in the previous seed development study utilizing the same lines as well as in other germination studies on *lpa* soybeans (Redekar et al., 2017; Yuan et al., 2017; Yu et al., 2019). Hence, because ABA has a major influence on seed dormancy and germination, its perturbed function in *lpa* soybean seeds may significantly contribute to the poor seed germination associated with these mutations. Consequently, the relationship between seed phytic acid content and ABA signaling warrants further investigation.

### Applying Results to Engineering or Breeding Enhanced Performance in *lpa* Crops

The new knowledge reported here of the transcriptome impacts during seed germination of various combinations of mutations in the *mips1* and *mrp* genes should prove invaluable both in understanding the molecular basis of the observed negative pleiotropic effects but also in identifying gene targets for engineering reduced negative effects, while maintaining desirable seed chemistry phenotypes. Taken together, the work reported here along with that of Yuan et al. (2017) illustrate the complexity of the downstream impacts on seed function of *mips*, *mrp*, and other *lpa* mutations. Broad impacts on P homeostasis, inositol phosphate signaling, starch metabolism, photosynthesis pathways, ABA signaling, and stress response make picking individual targets for engineering to reduce negative effects and restore nominal function challenging. In light of this complexity, traditional plant breeding approaches, such as recurrent selection for performance and yield within *lpa* lines or populations, as a compliment to targeted gene engineering, might prove effective in reducing these impacts, as concluded by Raboy (2020) and Colombo et al. (2020). An excellent example of this is illustrated by the work of Yuan et al. (2017). A single plant within a soybean *mips* population was identified that retained the *mips* mutation and *lpa* trait, but that had reduced negative effects on seed viability and germination. The molecular or genetic basis for this modification of the negative effects of the *mips* mutation has not yet been described.

While *mrp* mutations are effective in reducing seed phytic acid, in light of the many negative downstream effects previously reported and further elucidated by the transcriptome analyses reported here, perhaps use of alternative gene targets that have less impact on plant and seed performance would be recommended (Raboy, 2009a). An example of this would be a putative soybean ortholog of the barley and rice SPDT genes, which encodes a phosphorus transporter important to phytic acid synthesis (Raboy et al., 2015; Yamaji et al., 2017). Perturbations in these genes reduce seed phytic acid and seed total phosphorus but have little negative impact on plant and seed function or yield.

In contrast to the *mrp* mutations, *mips* mutations, in addition to reducing seed phytic acid also result in reduced raffinosaccharides and increased sucrose, a unique and highly desirable soybean seed phenotype (Saghai Maroof and Buss, 2011; Redekar et al., 2020). Therefore, there is added value in developing soybean *mips* lines with good field performance and yield. In addition to traditional breeding, methods for highly targeted gene engineering are advancing and this approach might prove valuable in the case of the *mips* mutations. An example that supports this possible approach is that described by Lee et al. (2015). This first case of engineering of a gene to serve as a modifier of poor fertility and growth of an *lpa* mutant targeted the *Arabidopsis* “Gle mRNA export factor.” The negative effects on vegetative growth and fertility observed in low-seed phytate *Arabidopsis* lines conditioned by knock-out of its Ipk1 (inositol pentakisphosphate 2-kinase) gene were rescued by engineering allelic variants of the Gle mRNA export factor for elevated Ins P6 sensitivity. The challenge then would be to use the data obtained here and in other similar studies to identify one or a few genes engineering of which would reduce the negative downstream effects of *mips* mutations.

## Conclusion

Disruption of phytic acid synthesis and accumulation elicited stress responses in the *mips1* and *mrp-l*/*mrp-n* mutants during seed germination. In addition to direct effects of reduced phytic acid, another origin of this stress is altered Pi homeostasis due to increased cellular Pi content and altered cellular *myo*-inositol content, which is diminished in *1mlpa* and *3mlpa* and increased in *2mlpa*. The downstream implications of these changes in Pi and *myo*-inositol content are manifold and could easily be cause for stress, thereby affecting normal cell functioning, seed viability, and ultimately germination and emergence potential. How this relates to the altered regulation in ABA signaling observed in all three *lpa* lines remains to be seen, but the changes observed in ABA signaling are significant, as ABA is a primary regulator of seed dormancy and inhibits germination. These findings, as well as findings in previous studies, indicate changes in ABA signaling may also interfere with germination potential in *lpa* seeds.

The broadness and complexity of the negative pleiotropic effects of these mutations indicate that targeted engineering to reduce these effects might prove challenging. While efforts at such targeted engineering should proceed, these results support the contention that traditional plant breeding within *lpa* lines and populations for plant and seed performance and yield would be a valuable compliment to targeted engineering in the effort to develop high-yielding *lpa* lines.

Lastly, to establish how the discovered biological processes are differentially regulated in the *lpa* lines used in this study, the GRNs constructed for each subset of genotypes are publicly available, consisting of interactions between TF genes and the target genes they putatively regulate. These interactions aim to help clarify the differential regulation of germination in *lpa* soybean seeds.

## Data Availability Statement

The datasets presented in this study can be found in online repositories. The names of the repository/repositories and accession number(s) can be found in the article/Supplementary Material.

## Author Contributions

LD and MS designed the experiment with SL’s participation. LD performed the sequencing experiments and analyzed the data and prepared the original draft of the manuscript. LD, MS, SL, and VR reviewed, edited, and finalized the paper. All authors contributed to the article and approved the submitted version.

## Conflict of Interest

The authors declare that the research was conducted in the absence of any commercial or financial relationships that could be construed as a potential conflict of interest.

## Publisher’s Note

All claims expressed in this article are solely those of the authors and do not necessarily represent those of their affiliated organizations, or those of the publisher, the editors and the reviewers. Any product that may be evaluated in this article, or claim that may be made by its manufacturer, is not guaranteed or endorsed by the publisher.

## References

[ref1] Banf M. Rhee S. Y. (2017). Computational inference of gene regulatory networks: approaches, limitations and opportunities. Biochim. Biophys. Acta Gene Regul. Mech. 1860, 41–52. 10.1016/j.bbagrm.2016.09.003, PMID: 27641093

[ref2] Bellieny-Rabelo D. De Oliveira E. A. Ribeiro E. S. Costa E. P. Oliveira A. E. Venancio T. M. (2016). Transcriptome analysis uncovers key regulatory and metabolic aspects of soybean embryonic axes during germination. Sci. Rep. 6:36009. 10.1038/srep36009 27824062 PMC5099898

[ref3] Bewley J. D. (1997). Seed germination and dormancy. Plant Cell 9, 1055–1066. 10.1105/tpc.9.7.1055, PMID: 12237375 PMC156979

[ref4] Blackman S. A. Obendorf R. L. Leopold A. C. (1995). Desiccation tolerance in developing soybean seeds—the role of stress proteins. Physiol. Plant. 93, 630–638. 10.1111/j.1399-3054.1995.tb05110.x PMC1075542 16652951

[ref5] Bohn L. Meyer A. S. Rasmussen S. K. (2008). Phytate: impact on environment and human nutrition. A challenge for molecular breeding. J. Zhejiang Univ. Sci. B 9, 165–191. 10.1631/jzus.B0710640, PMID: 18357620 PMC2266880

[ref6] Bove J. Jullien M. Grappin P. (2002). Functional genomics in the study of seed germination. Genome Biol. 3:reviews1002. 10.1186/gb-2001-3-1-reviews1002, PMID: 11806832 PMC150456

[ref7] Bowen D. E. Souza E. J. Guttieri M. J. Raboy V. Fu J. (2007). A low phytic acid barley mutation alters seed gene expression. Crop Sci. 47, S-149–S-159. 10.2135/cropsci2006.07.0456tpg

[ref8] Bray E. A. Beachy R. N. (1985). Regulation by ABA of beta-conglycinin expression in cultured developing soybean cotyledons. Plant Physiol. 79, 746–750. 10.1104/pp.79.3.746, PMID: 16664485 PMC1074964

[ref9] Bregitzer P. Raboy V. (2006). Effects of four independent low-phytate mutations on barley agronomic performance. Crop Sci. 46, 1318–1322. 10.2135/cropsci2005.09-0301

[ref10] Brown K. H. Solomons N. W. (1991). Nutritional problems of developing countries. Infect. Dis. Clin. N. Am. 5, 297–317. 10.1016/S0891-5520(20)30739-X, PMID: 1869811

[ref11] Campion B. Sparvoli F. Doria E. Tagliabue G. Galasso I. Fileppi M.. (2009). Isolation and characterisation of an *lpa* (low phytic acid) mutant in common bean (*Phaseolus vulgaris* L.). Theor. Appl. Genet. 118, 1211–1221. 10.1007/s00122-009-0975-8, PMID: 19224193

[ref12] Chen J. G. Pandey S. Huang J. Alonso J. M. Ecker J. R. Assmann S. M.. (2004). GCR1 can act independently of heterotrimeric G-protein in response to brassinosteroids and gibberellins in *Arabidopsis* seed germination. Plant Physiol. 135, 907–915. 10.1104/pp.104.038992, PMID: 15181210 PMC514125

[ref13] Cheng Y. Zhou W. El Sheery N. I. Peters C. Li M. Wang X.. (2011). Characterization of the *Arabidopsis* glycerophosphodiester phosphodiesterase (GDPD) family reveals a role of the plastid-localized AtGDPD1 in maintaining cellular phosphate homeostasis under phosphate starvation. Plant J. 66, 781–795. 10.1111/j.1365-313X.2011.04538.x, PMID: 21323773

[ref14] Chiera J. M. Finer J. J. Grabau E. A. (2004). Ectopic expression of a soybean phytase in developing seeds of *Glycine max* to improve phosphorus availability. Plant Mol. Biol. 56, 895–904. 10.1007/s11103-004-5293-6, PMID: 15821988

[ref15] Colombo F. Paolo D. Cominelli E. Sparvoli F. Nielsen E. Pilu R. (2020). MRP transporters and *low phytic acid* mutants in major crops: main pleiotropic effects and future perspectives. Front. Plant Sci. 11:1301. 10.3389/fpls.2020.01301, PMID: 32973854 PMC7481554

[ref16] Cutler S. R. Rodriguez P. L. Finkelstein R. R. Abrams S. R. (2010). Abscisic acid: emergence of a core signaling network. Annu. Rev. Plant Biol. 61, 651–679. 10.1146/annurev-arplant-042809-112122, PMID: 20192755

[ref17] del Pozo J. C. Allona I. Rubio V. Leyva A. de la Pena A. Aragoncillo C.. (1999). A type 5 acid phosphatase gene from *Arabidopsis thaliana* is induced by phosphate starvation and by some other types of phosphate mobilising/oxidative stress conditions. Plant J. 19, 579–589. 10.1046/j.1365-313X.1999.00562.x, PMID: 10504579

[ref18] DeMers L. C. Redekar N. R. Kachroo A. Tolin S. A. Li S. Saghai Maroof M. A. (2020). A transcriptional regulatory network of *Rsv3*-mediated extreme resistance against *soybean mosaic virus*. PLoS One 15:e0231658. 10.1371/journal.pone.0231658, PMID: 32315334 PMC7173922

[ref19] Dobin A. Davis C. A. Schlesinger F. Drenkow J. Zaleski C. Jha S.. (2013). STAR: ultrafast universal RNA-seq aligner. Bioinformatics 29, 15–21. 10.1093/bioinformatics/bts635, PMID: 23104886 PMC3530905

[ref20] Donahue J. L. Alford S. R. Torabinejad J. Kerwin R. E. Nourbakhsh A. Ray W. K.. (2010). The *Arabidopsis thaliana myo*-inositol 1-phosphate synthase1 gene is required for *myo*-inositol synthesis and suppression of cell death. Plant Cell 22, 888–903. 10.1105/tpc.109.071779, PMID: 20215587 PMC2861443

[ref21] Fait A. Angelovici R. Less H. Ohad I. Urbanczyk-Wochniak E. Fernie A. R.. (2006). *Arabidopsis* seed development and germination is associated with temporally distinct metabolic switches. Plant Physiol. 142, 839–854. 10.1104/pp.106.086694, PMID: 16963520 PMC1630763

[ref22] Faith J. J. Hayete B. Thaden J. T. Mogno I. Wierzbowski J. Cottarel G.. (2007). Large-scale mapping and validation of *Escherichia coli* transcriptional regulation from a compendium of expression profiles. PLoS Biol. 5:e8. 10.1371/journal.pbio.0050008, PMID: 17214507 PMC1764438

[ref23] Finkelstein R. R. Tenbarge K. M. Shumway J. E. Crouch M. L. (1985). Role of ABA in maturation of rapeseed embryos. Plant Physiol. 78, 630–636. 10.1104/pp.78.3.630, PMID: 16664296 PMC1064789

[ref24] Fisher R. A. (1992). “Statistical methods for research workers,” in Breakthroughs in Statistics. eds. Kotz S. Johnson N. L. (New York, NY: Springer New York), 66–70.

[ref25] Freed C. Adepoju O. Gillaspy G. (2020). Can inositol pyrophosphates inform strategies for developing low phytate crops? Plan. Theory 9:115. 10.3390/plants9010115, PMID: 31963418 PMC7020182

[ref26] Gilman A. G. (1987). G proteins: transducers of receptor-generated signals. Annu. Rev. Biochem. 56, 615–649. 10.1146/annurev.bi.56.070187.003151, PMID: 3113327

[ref27] Grant D. Nelson R. T. Cannon S. B. Shoemaker R. C. (2010). SoyBase, the USDA-ARS soybean genetics and genomics database. Nucleic Acids Res. 38, D843–D846. 10.1093/nar/gkp798, PMID: 20008513 PMC2808871

[ref28] Guttieri M. Bowen D. Dorsch J. A. Raboy V. Souza E. (2004). Identification and characterization of a low phytic acid wheat. Crop Sci. 44, 418–424. 10.2135/cropsci2004.4180

[ref29] Hampton J. G. TeKrony D. M. (1995). Handbook of Vigour Test Methods. Zurich, Switzerland: The International Seed Testing Association.

[ref30] Haque S. Ahmad J. S. Clark N. M. Williams C. M. Sozzani R. (2019). Computational prediction of gene regulatory networks in plant growth and development. Curr. Opin. Plant Biol. 47, 96–105. 10.1016/j.pbi.2018.10.005, PMID: 30445315

[ref31] Haury A. C. Mordelet F. Vera-Licona P. Vert J. P. (2012). TIGRESS: trustful inference of gene regulation using stability selection. BMC Syst. Biol. 6:145. 10.1186/1752-0509-6-145, PMID: 23173819 PMC3598250

[ref32] Hecker M. Lambeck S. Toepfer S. van Someren E. Guthke R. (2009). Gene regulatory network inference: data integration in dynamic models—a review. Biosystems 96, 86–103. 10.1016/j.biosystems.2008.12.004, PMID: 19150482

[ref33] Hitz W. D. Carlson T. J. Kerr P. S. Sebastian S. A. (2002). Biochemical and molecular characterization of a mutation that confers a decreased raffinosaccharide and phytic acid phenotype on soybean seeds. Plant Physiol. 128, 650–660. 10.1104/pp.010585, PMID: 11842168 PMC148927

[ref34] Horbowicz M. Obendorf R. L. (1994). Seed desiccation tolerance and storability: dependence on flatulence-producing oligosaccharides and cyclitols-review and survey. Seed Sci. Res. 4, 385–405. 10.1017/S0960258500002440

[ref35] Howell K. A. Narsai R. Carroll A. Ivanova A. Lohse M. Usadel B.. (2009). Mapping metabolic and transcript temporal switches during germination in rice highlights specific transcription factors and the role of RNA instability in the germination process. Plant Physiol. 149, 961–980. 10.1104/pp.108.129874, PMID: 19074628 PMC2633829

[ref36] Hubbard K. E. Nishimura N. Hitomi K. Getzoff E. D. Schroeder J. I. (2010). Early abscisic acid signal transduction mechanisms: newly discovered components and newly emerging questions. Genes Dev. 24, 1695–1708. 10.1101/gad.1953910, PMID: 20713515 PMC2922499

[ref37] Huynh-Thu V. A. Irrthum A. Wehenkel L. Geurts P. (2010). Inferring regulatory networks from expression data using tree-based methods. PLoS One 5:e12776. 10.1371/journal.pone.0012776, PMID: 20927193 PMC2946910

[ref38] Israel D. W. Taliercio E. Kwanyuen P. Burton J. W. Dean L. (2011). Inositol metabolism in developing seed of low and normal phytic acid soybean lines. Crop Sci. 51, 282–289. 10.2135/cropsci2010.03.0123

[ref39] Jensen M. K. Lindemose S. de Masi F. Reimer J. J. Nielsen M. Perera V.. (2013). ATAF1 transcription factor directly regulates abscisic acid biosynthetic gene NCED3 in *Arabidopsis thaliana*. FEBS Open Bio 3, 321–327. 10.1016/j.fob.2013.07.006, PMID: 23951554 PMC3741915

[ref40] Jiang H. Lei R. Ding S. W. Zhu S. (2014). Skewer: a fast and accurate adapter trimmer for next-generation sequencing paired-end reads. BMC Bioinform. 15:182. 10.1186/1471-2105-15-182, PMID: 24925680 PMC4074385

[ref41] Jin J. Tian F. Yang D. C. Meng Y. Q. Kong L. Luo J.. (2017). PlantTFDB 4.0: toward a central hub for transcription factors and regulatory interactions in plants. Nucleic Acids Res. 45, D1040–D1045. 10.1093/nar/gkw982, PMID: 27924042 PMC5210657

[ref42] Kagaya Y. Okuda R. Ban A. Toyoshima R. Tsutsumida K. Usui H.. (2005). Indirect ABA-dependent regulation of seed storage protein genes by FUSCA3 transcription factor in *Arabidopsis*. Plant Cell Physiol. 46, 300–311. 10.1093/pcp/pci031, PMID: 15695463

[ref43] Kim S. I. Tai T. H. (2011). Identification of genes necessary for wild-type levels of seed phytic acid in *Arabidopsis thaliana* using a reverse genetics approach. Mol. Gen. Genomics. 286, 119–133. 10.1007/s00438-011-0631-2, PMID: 21698461

[ref44] Krouk G. Lingeman J. Colon A. M. Coruzzi G. Shasha D. (2013). Gene regulatory networks in plants: learning causality from time and perturbation. Genome Biol. 14:123. 10.1186/gb-2013-14-6-123 23805876 PMC3707030

[ref45] Kruger N. J. von Schaewen A. (2003). The oxidative pentose phosphate pathway: structure and organisation. Curr. Opin. Plant Biol. 6, 236–246. 10.1016/S1369-5266(03)00039-6, PMID: 12753973

[ref46] Kuo H. F. Hsu Y. Y. Lin W. C. Chen K. Y. Munnik T. Brearley C. A.. (2018). *Arabidopsis* inositol phosphate kinases IPK1 and ITPK1 constitute a metabolic pathway in maintaining phosphate homeostasis. Plant J. 95, 613–630. 10.1111/tpj.13974 29779236

[ref47] Larson S. R. Rutger J. N. Young K. A. Raboy V. (2000). Isolation and genetic mapping of a non-lethal rice (*Oryza sativa* L.) *low phytic acid 1* mutation. Crop Sci. 40, 1397–1405. 10.2135/cropsci2000.4051397x

[ref48] Larson S. R. Young K. A. Cook A. Blake T. K. Raboy V. (1998). Linkage mapping of two mutations that reduce phytic acid content of barley grain. Theor. Appl. Genet. 97, 141–146. 10.1007/s001220050878

[ref49] Lee H. S. Lee D. H. Cho H. K. Kim S. H. Auh J. H. Pai H. S. (2015). InsP6-sensitive variants of the Gle1 mRNA export factor rescue growth and fertility defects of the *ipk1* low-phytic-acid mutation in *Arabidopsis*. Plant Cell 27, 417–431. 10.1105/tpc.114.132134, PMID: 25670768 PMC4456929

[ref50] Li Y. Pearl S. A. Jackson S. A. (2015). Gene networks in plant biology: approaches in reconstruction and analysis. Trends Plant Sci. 20, 664–675. 10.1016/j.tplants.2015.06.013, PMID: 26440435

[ref51] Liao Y. Smyth G. K. Shi W. (2014). featureCounts: an efficient general purpose program for assigning sequence reads to genomic features. Bioinformatics 30, 923–930. 10.1093/bioinformatics/btt656, PMID: 24227677

[ref52] Liu X. Zhang H. Zhao Y. Feng Z. Li Q. Yang H. Q.. (2013). Auxin controls seed dormancy through stimulation of abscisic acid signaling by inducing ARF-mediated ABI3 activation in *Arabidopsis*. Proc. Natl. Acad. Sci. U. S. A. 110, 15485–15490. 10.1073/pnas.1304651110 23986496 PMC3780901

[ref53] Loewus F. A. Chen M.-S. Loewus M. W. (1973). “The myo-inositol oxidation pathway to cell wall polysaccharides,” in Biogenesis of Plant Cell Wall Polysaccharides. ed. Loewus F. A. (New York: Academic Press, Inc.), 1–28.

[ref54] Loewus F. A. Murthy P. P. N. (2000). *Myo*-inositol metabolism in plants. Plant Sci. 150, 1–19. 10.1016/S0168-9452(99)00150-8

[ref55] Lopez-Molina L. Mongrand S. Chua N. H. (2001). A postgermination developmental arrest checkpoint is mediated by abscisic acid and requires the ABI5 transcription factor in *Arabidopsis*. Proc. Natl. Acad. Sci. U. S. A. 98, 4782–4787. 10.1073/pnas.081594298 11287670 PMC31911

[ref56] Lott J. N. A. Greenwood J. S. Batten G. D. (1995). “Mechanisms and regulation of mineral nutrient storage during seed development,” in Seed Development and Germination. eds. Kigel J. Galili G. (New York: Marcel Dekker Inc.), 215.

[ref57] Love M. I. Huber W. Anders S. (2014). Moderated estimation of fold change and dispersion for RNA-seq data with DESeq2. Genome Biol. 15:550. 10.1186/s13059-014-0550-8 25516281 PMC4302049

[ref58] Lu G. Paul A. L. McCarty D. R. Ferl R. J. (1996). Transcription factor veracity: is GBF3 responsible for ABA-regulated expression of *Arabidopsis* Adh? Plant Cell 8, 847–857. 10.1105/tpc.8.5.847, PMID: 8672884 PMC161143

[ref59] Luo Y. Qin G. Zhang J. Liang Y. Song Y. Zhao M.. (2011). D-*myo*-inositol-3-phosphate affects phosphatidylinositol-mediated endomembrane function in *Arabidopsis* and is essential for auxin-regulated embryogenesis. Plant Cell 23, 1352–1372. 10.1105/tpc.111.083337, PMID: 21505066 PMC3101546

[ref108] Marbach D. Costello J. C. Küffner R. Vega N. M. Prill R. J. Camacho D. M.. (2012). Wisdom of crowds for robust gene network inference. Nat. Methods 9, 796–804. 10.1038/nmeth.2016 22796662 PMC3512113

[ref60] Margolin A. A. Nemenman I. Basso K. Wiggins C. Stolovitzky G. Dalla Favera R.. (2006). ARACNE: an algorithm for the reconstruction of gene regulatory networks in a mammalian cellular context. BMC Bioinform. 7:S7. 10.1186/1471-2105-7-S1-S7 PMC1810318 16723010

[ref61] Meis S. J. Fehr W. R. Schnebly S. R. (2003). Seed source effect on field emergence of soybean lines with reduced phytate and raffinose saccharides. Crop Sci. 43, 1336–1339. 10.2135/cropsci2003.1336

[ref62] Murthy P. P. N. (1996). “Inositol phosphates and their metabolism in plants,” in Myo-Inositol Phosphates, Phosphoinositides, and Signal Transduction. eds. Biswas B. B. Biswas S. (Boston, MA: Springer US), 227–255. 10.1007/978-1-4613-0343-5_8 8744267

[ref63] Nagy R. Grob H. Weder B. Green P. Klein M. Frelet-Barrand A.. (2009). The *Arabidopsis* ATP-binding cassette protein AtMRP5/AtABCC5 is a high affinity inositol hexakisphosphate transporter involved in guard cell signaling and phytate storage. J. Biol. Chem. 284, 33614–33622. 10.1074/jbc.M109.030247, PMID: 19797057 PMC2785203

[ref64] Nunes A. C. Vianna G. R. Cuneo F. Amaya-Farfan J. de Capdeville G. Rech E. L.. (2006). RNAi-mediated silencing of the *myo*-inositol-1-phosphate synthase gene (GmMIPS1) in transgenic soybean inhibited seed development and reduced phytate content. Planta 224, 125–132. 10.1007/s00425-005-0201-0, PMID: 16395584

[ref65] Obendorf R. L. (1997). Oligosaccharides and galactosyl cyclitols in seed desiccation tolerance. Seed Sci. Res. 7, 63–74. 10.1017/S096025850000341X

[ref66] Obendorf R. L. Zimmerman A. D. Zhang Q. Y. Castillo A. Kosina S. M. Bryant E. G.. (2009). Accumulation of soluble carbohydrates during seed development and maturation of low-raffinose, low-stachyose soybean. Crop Sci. 49, 329–341. 10.2135/cropsci2008.06.0370

[ref67] Oltmans S. E. Fehr W. R. Welke G. A. Raboy V. Peterson K. L. (2005). Agronomic and seed traits of soybean lines with low-phytate phosphorus. Crop Sci. 45, 593–598. 10.2135/cropsci2005.0593

[ref68] O’Malley R. C. Huang S. C. Song L. Lewsey M. G. Bartlett A. Nery J. R.. (2016). Cistrome and epicistrome features shape the regulatory DNA landscape. Cell 165, 1280–1292. 10.1016/j.cell.2016.04.038, PMID: 27203113 PMC4907330

[ref69] Pandey S. Assmann S. M. (2004). The *Arabidopsis* putative G protein-coupled receptor GCR1 interacts with the G protein alpha subunit GPA1 and regulates abscisic acid signaling. Plant Cell 16, 1616–1632. 10.1105/tpc.020321, PMID: 15155892 PMC490050

[ref70] Panzeri D. Cassani E. Doria E. Tagliabue G. Forti L. Campion B.. (2011). A defective ABC transporter of the MRP family, responsible for the bean *lpa1* mutation, affects the regulation of the phytic acid pathway, reduces seed *myo*-inositol and alters ABA sensitivity. New Phytol. 191, 70–83. 10.1111/j.1469-8137.2011.03666.x, PMID: 21395595

[ref71] Peterbauer T. Puschenreiter M. Richter A. (1998). Metabolism of galactosylononitol in seeds of *Vigna umbellata*. Plant Cell Physiol. 39, 334–341. 10.1093/oxfordjournals.pcp.a029374

[ref72] Peterbauer T. Richter A. (1998). Galactosylononitol and stachyose synthesis in seeds of adzuki bean. Plant Physiol. 117, 165–172. 10.1104/pp.117.1.165, PMID: 9576785 PMC34999

[ref73] Pilu R. Panzeri D. Gavazzi G. Rasmussen S. K. Consonni G. Nielsen E. (2003). Phenotypic, genetic and molecular characterization of a maize low phytic acid mutant (*lpa241*). Theor. Appl. Genet. 107, 980–987. 10.1007/s00122-003-1316-y, PMID: 14523526

[ref74] Raboy V. (2007). “Seed phosphorous and the development of low-phytate crops,” in Inositol Phosphates: Linking Agriculture and the Environment. eds. Richardson A. E. Mullaney E. J. (Oxfordshire, UK: CAB International), 111–132.

[ref75] Raboy V. (2009a). Approaches and challenges to engineering seed phytate and total phosphorus. Plant Sci. 177, 281–296. 10.1016/j.plantsci.2009.06.012

[ref76] Raboy V. (2009b). “Seed total phosphate and phytic acid,” in Molecular Genetic Approaches to Maize Improvement. eds. Kriz A. L. Larkins B. A. (Berlin, Heidelberg: Springer Berlin Heidelberg), 41–53.

[ref77] Raboy V. (2020). *Low phytic acid* crops: observations based on four decades of research. Plan. Theory 9:140. 10.3390/plants9020140, PMID: 31979164 PMC7076677

[ref78] Raboy V. Gerbasi P. F. Young K. A. Stoneberg S. D. Pickett S. G. Bauman A. T.. (2000). Origin and seed phenotype of maize low phytic acid 1-1 and low phytic acid 2-1. Plant Physiol. 124, 355–368. 10.1104/pp.124.1.355, PMID: 10982449 PMC59149

[ref79] Raboy V. Peterson K. Jackson C. Marshall J. M. Hu G. Saneoka H.. (2015). A substantial fraction of barley (*Hordeum vulgare* L.) low phytic acid mutations have little or no effect on yield across diverse production environments. Plan. Theory 4, 225–239. 10.3390/plants4020225, PMID: 27135325 PMC4844328

[ref80] Rajjou L. Duval M. Gallardo K. Catusse J. Bally J. Job C.. (2012). Seed germination and vigor. Annu. Rev. Plant Biol. 63, 507–533. 10.1146/annurev-arplant-042811-105550, PMID: 22136565

[ref81] Ravindran V. Bryden W. L. Kornegay E. T. (1995). Phytates: occurrence, bioavailability and implications in poultry nutrition. Poul. Avian Biol. Rev. 6, 125–143.

[ref82] Redekar N. R. Biyashev R. M. Jensen R. V. Helm R. F. Grabau E. A. Maroof M. A. (2015). Genome-wide transcriptome analyses of developing seeds from low and normal phytic acid soybean lines. BMC Genomics 16:1074. 10.1186/s12864-015-2283-9, PMID: 26678836 PMC4683714

[ref83] Redekar N. R. Glover N. M. Biyashev R. M. Ha B. Raboy V. Saghai Maroof M. A. (2020). Genetic interactions regulating seed phytate and oligosaccharides in soybean (*Glycine max* L.). PLoS One 15:e0235120. 10.1371/journal.pone.0235120, PMID: 32584851 PMC7316244

[ref84] Redekar N. Pilot G. Raboy V. Li S. Saghai Maroof M. A. (2017). Inference of transcription regulatory network in low phytic acid soybean seeds. Front. Plant Sci. 8:2029. 10.3389/fpls.2017.02029, PMID: 29250090 PMC5714895

[ref85] Saghai Maroof M. A. Buss G. R. (2011). *Low phytic acid, low stachyose, high sucrose soybean lines*. U.S. patent No 12/033,830. Inventors; Virginia Tech Intellectual Properties Inc., Assignee.

[ref86] Saghai Maroof M. A. Glover N. M. Biyashev R. M. Buss G. R. Grabau E. A. (2009). Genetic basis of the low-phytate trait in the soybean line CX1834. Crop Sci. 49, 69–76. 10.2135/cropsci2008.06.0362

[ref87] Schafer J. Strimmer K. (2005). An empirical Bayes approach to inferring large-scale gene association networks. Bioinformatics 21, 754–764. 10.1093/bioinformatics/bti062, PMID: 15479708

[ref88] Schmutz J. Cannon S. B. Schlueter J. Ma J. Mitros T. Nelson W.. (2010). Genome sequence of the palaeopolyploid soybean. Nature 463, 178–183. 10.1038/nature08670 20075913

[ref89] Schwarz G. (1978). Estimating the dimension of a model. Ann. Stat. 6, 461–464. 10.1214/aos/1176344136

[ref90] Scrucca L. Fop M. Murphy T. B. Raftery A. E. (2016). Mclust 5: clustering, classification and density estimation using Gaussian finite mixture models. R J. 8, 289–317. 10.32614/RJ-2016-021, PMID: 27818791 PMC5096736

[ref91] Segal E. Shapira M. Regev A. Pe’er D. Botstein D. Koller D.. (2003). Module networks: identifying regulatory modules and their condition-specific regulators from gene expression data. Nat. Genet. 34, 166–176. 10.1038/ng1165, PMID: 12740579

[ref92] Shewry P. R. Napier J. A. Tatham A. S. (1995). Seed storage proteins: structures and biosynthesis. Plant Cell 7, 945–956. 10.1105/tpc.7.7.945, PMID: 7640527 PMC160892

[ref93] Shi J. Wang H. Schellin K. Li B. Faller M. Stoop J. M.. (2007). Embryo-specific silencing of a transporter reduces phytic acid content of maize and soybean seeds. Nat. Biotechnol. 25, 930–937. 10.1038/nbt1322, PMID: 17676037

[ref94] Slovin J. P. Bandurski R. S. Cohen J. D. (1999). “Auxin,” in Biochemistry and Molecular Biology of Plant Hormones. eds. Hooykaas P. J. J. Hall M. A. Libbenga K. R. (Amsterdam, The Netherlands: Elsevier), 115–140.

[ref95] Tan X. Calderon-Villalobos L. I. Sharon M. Zheng C. Robinson C. V. Estelle M.. (2007). Mechanism of auxin perception by the TIR1 ubiquitin ligase. Nature 446, 640–645. 10.1038/nature05731, PMID: 17410169

[ref96] Theodorou M. E. Plaxton W. C. (1993). Metabolic adaptations of plant respiration to nutritional phosphate deprivation. Plant Physiol. 101, 339–344. 10.1104/pp.101.2.339, PMID: 12231689 PMC160576

[ref97] van Dijk E. L. Auger H. Jaszczyszyn Y. Thermes C. (2014). Ten years of next-generation sequencing technology. Trends Genet. 30, 418–426. 10.1016/j.tig.2014.07.001 25108476

[ref98] Veljanovski V. Vanderbeld B. Knowles V. L. Snedden W. A. Plaxton W. C. (2006). Biochemical and molecular characterization of AtPAP26, a vacuolar purple acid phosphatase up-regulated in phosphate-deprived *Arabidopsis* suspension cells and seedlings. Plant Physiol. 142, 1282–1293. 10.1104/pp.106.087171, PMID: 16963519 PMC1630754

[ref99] Walker D. R. Scaboo A. M. Pantalone V. R. Wilcox J. R. Boerma H. R. (2006). Genetic mapping of loci associated with seed phytic acid content in CX1834-1-2 soybean. Crop Sci. 46, 390–397. 10.2135/cropsci2005.0245

[ref100] Weaver C. M. Kannan S. (2001). “Phytate and mineral bioavailability,” in Food Phytates. eds. Reddy N. R. Sathe S. K. (Boca Raton, FL: CRC Press), 211–223.

[ref101] Wilcox J. R. Premachandra G. S. Young K. A. Raboy V. (2000). Isolation of high seed inorganic P, low-phytate soybean mutants. Crop Sci. 40, 1601–1605. 10.2135/cropsci2000.4061601x

[ref102] Wu Y. Deng Z. Lai J. Zhang Y. Yang C. Yin B.. (2009). Dual function of *Arabidopsis* ATAF1 in abiotic and biotic stress responses. Cell Res. 19, 1279–1290. 10.1038/cr.2009.108, PMID: 19752887

[ref103] Xu X. H. Zhao H. J. Liu Q. L. Frank T. Engel K. H. An G.. (2009). Mutations of the multi-drug resistance-associated protein ABC transporter gene 5 result in reduction of phytic acid in rice seeds. Theor. Appl. Genet. 119, 75–83. 10.1007/s00122-009-1018-1, PMID: 19370321

[ref104] Yamaji N. Takemoto Y. Miyaji T. Mitani-Ueno N. Yoshida K. T. Ma J. F. (2017). Reducing phosphorus accumulation in rice grains with an impaired transporter in the node. Nature 541, 92–95. 10.1038/nature20610, PMID: 28002408

[ref105] Yanhui C. Xiaoyuan Y. Kun H. Meihua L. Jigang L. Zhaofeng G.. (2006). The MYB transcription factor superfamily of *Arabidopsis*: expression analysis and phylogenetic comparison with the rice MYB family. Plant Mol. Biol. 60, 107–124. 10.1007/s11103-005-2910-y, PMID: 16463103

[ref106] Yu X. Jin H. Fu X. Yang Q. Yuan F. (2019). Quantitative proteomic analyses of two soybean low phytic acid mutants to identify the genes associated with seed field emergence. BMC Plant Biol. 19:569. 10.1186/s12870-019-2201-4 31856712 PMC6921446

[ref107] Yuan F. Yu X. Dong D. Yang Q. Fu X. Zhu S.. (2017). Whole genome-wide transcript profiling to identify differentially expressed genes associated with seed field emergence in two soybean low phytate mutants. BMC Plant Biol. 17:16. 10.1186/s12870-016-0953-7, PMID: 28100173 PMC5242038

